# Avian Influenza in Wild Birds and Poultry: Dissemination Pathways, Monitoring Methods, and Virus Ecology

**DOI:** 10.3390/pathogens10050630

**Published:** 2021-05-20

**Authors:** Artem Blagodatski, Kseniya Trutneva, Olga Glazova, Olga Mityaeva, Liudmila Shevkova, Evgenii Kegeles, Nikita Onyanov, Kseniia Fede, Anna Maznina, Elena Khavina, Seon-Ju Yeo, Hyun Park, Pavel Volchkov

**Affiliations:** 1 Life Sciences Research Center, Moscow Institute of Physics and Technology, 141700 Dolgoprudniy, Russia; trutneva-k@mail.ru (K.T.); ol6ga@mail.ru (O.M.); Luda4everandever@gmail.com (L.S.); kegeles.ea@phystech.edu (E.K.); onyanov.na@phystech.edu (N.O.); fede.kv@phystech.edu (K.F.); aamaznina@gmail.com (A.M.); khavina.lx@phystech.edu (E.K.); vpwwww@gmail.com (P.V.); 2 Institute of Theoretical and Experimental Biophysics, Russian Academy of Sciences Pushchino, 142290 Moscow, Russia; 3 Research Institute of Personalized Medicine, National Center for Personalized Medicine of Endocrine Diseases, The National Medical Research Center for Endocrinology, 117036 Moscow, Russia; 4 Department of Tropical Medicine and Parasitology, College of Medicine, Seoul National University, Seoul 03080, Korea; yeosj@snu.ac.kr; 5 Zoonosis Research Center, Department of Infection Biology, School of Medicine, Wonkwang University, Iksan 570-749, Korea; hyunpk@wku.ac.kr

**Keywords:** avian influenza, influenza dissemination, influenza strains, influenza monitoring, influenza ecology, avian influenza management

## Abstract

Avian influenza is one of the largest known threats to domestic poultry. Influenza outbreaks on poultry farms typically lead to the complete slaughter of the entire domestic bird population, causing severe economic losses worldwide. Moreover, there are highly pathogenic avian influenza (HPAI) strains that are able to infect the swine or human population in addition to their primary avian host and, as such, have the potential of being a global zoonotic and pandemic threat. Migratory birds, especially waterfowl, are a natural reservoir of the avian influenza virus; they carry and exchange different virus strains along their migration routes, leading to antigenic drift and antigenic shift, which results in the emergence of novel HPAI viruses. This requires monitoring over time and in different locations to allow for the upkeep of relevant knowledge on avian influenza virus evolution and the prevention of novel epizootic and epidemic outbreaks. In this review, we assess the role of migratory birds in the spread and introduction of influenza strains on a global level, based on recent data. Our analysis sheds light on the details of viral dissemination linked to avian migration, the viral exchange between migratory waterfowl and domestic poultry, virus ecology in general, and viral evolution as a process tightly linked to bird migration. We also provide insight into methods used to detect and quantify avian influenza in the wild. This review may be beneficial for the influenza research community and may pave the way to novel strategies of avian influenza and HPAI zoonosis outbreak monitoring and prevention.

## 1. Introduction

The influenza virus is known to be common in avians and mammals, including humans. Its strains are commonly characterized by a combination pattern of two surface proteins, hemagglutinin (HA) and neuraminidase (NA). The viral genome consists of eight highly mutable RNA molecules which continuously undergo mutation and shuffling while infecting the host genome, giving rise to novel viral strains capable of evading existing host immunity [1]. Both point mutations in key epitopes known as antigenic drift [2] and antigenic shift between viruses from two or more hosts infecting the same host and reassembling [3] can generate a novel life-threatening virus which is no longer recognized by previously developed antibodies within human, swine, and domestic fowl populations, leading to them acquiring pandemic potential.

Avian influenza viruses (AIV) are responsible for a sharp increase in the number of outbreaks of this disease in poultry and other birds in recent years. Several of the AI virus subtypes, in particular H5, H7, and H9, have shown their ability to cross the species barrier and infect mammals, including swine and humans [4]. Therefore, avian influenza is a major concern for public health. Highly pathogenic avian influenza (HPAI) viruses are primarily composed of H5 and H7 subtypes, and infection with these viruses may result in 100% mortality within a susceptible poultry species. Low pathogenic AI (LPAI) viruses can result in asymptomatic infection or manifest with milder signs of respiratory infection. It should be noted, however, that LPAI refers specifically to disease manifestation in poultry, as there have been LPAIs, such as H7N9 LPAI, that have caused severe infection, and even death, in humans. Viral reassortment is the source of emergence of deadly HPAI viruses that are transmissible to mammals and are prone to adaptive evolution in their new hosts [2]. Novel HPAI strains have previously led to mass epizootic outbreaks, as well as human-threatening epidemics [5,6]. It is still disputed whether the infamous 1918 Spanish flu pandemic was caused by a reassortant strain evolved in mammals [7] or whether it was an entirely avian-like virus that adapted to humans [8]. Worldwide, migratory bird populations are both a vast reservoir of influenza virus and a playground where novel, potentially highly pathogenic, strains of AIV emerge [5]. Primary avian migration routes can be regarded as a network interconnecting the entire global population of influenza virus. It is highly important that the diversity of the current epizootic situation in wild migratory birds is continuously monitored worldwide [9]. It provides the necessary information for monitoring the influenza virus landscape for potential pandemic viruses, which enables the prevention of novel outbreaks and enables the yearly update of current seasonal influenza vaccines. Information on the genome sequences of circulating AIV strains also has potential for use in the development of next-generation vaccines, utilizing approaches such as reverse genetics or DNA vaccination [10]. While not as devastating as HPAI, low pathogenic avian influenza (LPAI) strains represent a permanently existing reservoir of viral diversity from where novel strains continuously emerge. Thus, LPAIV circulation in wild birds poses an indirect threat to poultry and humans, and it is critical to understand the ecology of LPAIV to prevent disease in these populations.

In this review, we analyze HPAI and LPAI introduction caused by wild bird migration, as well as the stability, persistence, and infective potential of AIV in the environment. We also discuss existing methods of influenza virus monitoring and detection in wild migratory bird populations, as well as modeling methods of virus dissemination. We also touch on the AIV exchange between migratory waterfowl, land birds, and domestic poultry. By summarizing our collective knowledge on avian influenza as linked to avian migration, we intend to assist in the prevention of harmful viral outbreaks in the future.

## 2. Brief Overview of Epizootic and Zoonotic Outbreaks of the Avian Influenza Virus

The wild migratory bird population is a vast natural viral reservoir that regularly gives rise to novel influenza A strains capable of infecting humans and causing epidemics. In the 20th and 21st centuries, there were several notable and well-documented outbreaks of avian influenza that led to human fatalities. The most paramount ones are detailed below, according to their strain.

During the 1997 Hong Kong outbreak of the H5N1 subtype virus, humans were infected directly from chickens, without the involvement of an intermediate host [6,11,12], which resulted in 18 cases of diseased humans, 6 of which led to death. The further spread of the zoonosis was prevented by means of territory-wide poultry slaughter, with more than 1.5 million chickens slaughtered by the end of December 1997. Despite having an apparently high fatality rate—though it should be noted that this rate is thought to be related to the underreporting of the disease, especially in mildly symptomatic individuals—and being extremely virulent to humans, the outbreak failed to acquire features associated with viruses of pandemic potential, namely rapid spread to all areas of the world, resulting in an unusually high instance of illness and death for approximately 2 to 3 years [13]. The hemagglutinin gene of the Hong Kong 1997 H5N1 virus highly likely emerged from the influenza A/goose/Guangdong/1/96 (H5N1) isolated from a sick goose during an outbreak in Guangdong province, China, in 1996. Another parent strain that was involved in the reassortment that gave rise to the 1997 H5N1 strain was the A/quail/Hong Kong/G1/97 (H9N2) strain, as it contained the internal genes PB1 and PB2, which were highly similar to the 1997 H5N1. Another likely parent could be the A/teal/Hong Kong/W312/97 (H6N1)-related virus, as it carried six internal genes alongside the neuraminidase gene, all of which were highly homologous to the Hong Kong virus of 1997. Taken together, these data give us an image of aquatic birds as a viral reservoir where different strains cocirculate, reassort, and, occasionally, lead to the emergence of novel highly pathogenic strains, such as the low pathogenic H5N1, H9N2, and H6N1 influenza strains that were circulating in waterfowl between 1996 and 1997 and recombined into the 1997 HPAI zoonotic virus [12]. The H5N1 Hong Kong virus re-emerged in subsequent years in Southeastern Asia. In December 2002 and January 2003, new cases were reported in Hong Kong in both poultry and wild birds. In late 2003 and early 2004, a series of outbreaks occurred in South Korea and Indonesia, later spreading to Vietnam, Japan, Taiwan, Cambodia, Laos, Thailand, and mainland China [13]. H5N1 is the most prevalent strain present on the African continent, with 13 of 53 surveyed countries reporting HPAI in poultry and with a further three instances reported in wild birds between 2003 and 2005; all but one of the reported outbreaks was H5N1 [14].

Another avian influenza subtype, namely H7N9, broke out in China in March 2013, infecting humans in a wave-like manner for five years until 2017. The virus originated as an LPAIV, only found in chickens following active surveillance after viral isolate in infected humans was analyzed, leading to suspicion of its avian origin. To date, according to WHO data, a total of 916 laboratory-confirmed human infections with avian influenza A (H7N9) virus have been reported through IHR notification since early 2013 [15]. The previously mentioned H7N9 outbreak in China was caused by a novel virus subtype that contained H9N2 internal genes, which were acquired during constant reassortments in poultry markets. This zoonotic infection became one of the most severe AIV outbreaks, causing high morbidity and case fatality (40.6%) in humans [16].

Outbreaks of an AI strain of lower pathogenicity, H7N3, occur regularly, dealing great damage to both poultry farmers and the industry. There have been several significant instances in recent years, including the outbreaks in Italy [17] and Chile [18] in 2004, with the latter case bringing the emergence of an HPAI strain from an LPAI precursor due to a 30-nucleotide insertion in the hemagglutinin cleavage site. The H7N3 strain has also been reported to be pathogenic to humans, as it was in British Columbia, Canada, in 2004 [18], where a novel insertion in the hemagglutinin cleavage site of the human and HPAI isolate occurred, likely caused by recombination between the hemagglutination and matrix genes in the LPAI virus [19]. Taking this into account, it can be proposed that the HA cleavage site is a highly mutable region responsible for the generation of novel virulent influenza strains.

Outbreaks of H7N7 HPAI strains in the early 1980s demonstrated the ability to jump over from birds to harbor seals, resulting in mass mortality of the latter in 1979 and 1980 [20], thus showing interspecies transmission potential and being an important research object as a strain potentially dangerous for mammals, including humans. The H9N2 strain circulating in Hong Kong at the same time as the H7N7 strain could be the donor of its PB1 and PB2 internal gene segments [21]. Indeed, the H9N2 strain has shown infectious potential in humans, with at least two confirmed cases in Hong Kong in 1999 [22], and it is recognized as a pandemic threat [23], so constant monitoring of avian-exposed human populations for influenza A (H9N2) infection is highly recommended for preventive purposes.

Genetic studies have revealed that the influenza A H2N2 virus that caused the Asian influenza pandemic of 1957 derived its hemagglutinin, neuraminidase, and polymerase (PB1) genes from an avian influenza A virus [24]. This pandemic strain is therefore thought to have evolved as a result of genetic recombination between an avian influenza A virus and the circulating human influenza A (H1N1) virus.

Similarly, the H3N2 virus that caused the Hong Kong pandemic of 1968 was a reassortant virus that acquired its hemagglutinin and PB1 genes from an avian influenza virus and remaining gene segments from the circulating H2N2 virus [24]. Other HPAI strains reported to cause outbreaks in 343 and 76 poultry farms in 2016 and 2017, respectively, without affecting the human population, were from the H5N6 and H5N8 subtypes. These outbreaks led to an unprecedented level of damage to the poultry industry in Korea: 38 million birds were killed, resulting in vast financial losses (approximately USD 312 million). AI vaccination in conjunction with surveillance and depopulation was required by some poultry producers and animal welfare organizations [25]. A review of recent avian influenza outbreaks allows us to estimate the overall frequency and scale of the problem. Between November 2019 and February 2020, 37 H5N8 HPAI virus outbreaks were reported in poultry (34 cases), captive birds (1 case), and wild birds (2 cases) in Europe alone [26]. Genomic characterization of the previously discussed H5N8 strain that emerged in Europe was evaluated as reassortant of H5N8 HPAI strains from Africa and LPAI strains from Eurasia. The reassortment likely occurred in wild migratory birds in Asia during the summer and then spread to Eastern Europe with the autumn migration, thus being the first wild bird migration from Africa to Eurasia implicated in the long-distance spread of HPAI viruses to the EU [26]. There has been a recent re-emergence of this strain on a poultry farm in South Africa, in April 2021 [27].

The H5N6 and H5N8 HPAI strains have also shown the potential to infect the human population. Thirty-one human cases of A(H5N6) have been reported in China to date (since 2014) [28]. A(H5N8) has caused seven human infections in Russia in early 2021 without sustained human-to-human transmission [29]

While this review does not mention all reported HPAI and LPAI outbreaks, it presents enough to provide a snapshot of the nature of the seemingly instantaneous contact and recombination of the avian and mammalian influenza viral pools; this, in turn, gives continuous rise to novel pathogenic strains, including those with pandemic potential. Zoonotic, pandemic, and devastating epizootic strains typically arise as reassortants from LPAI viruses circulating in poultry and in the wild bird population. To preempt and prevent outbreaks of pandemic potential, the evolution and diversity of avian influenza viruses require constant monitoring for preventative action.

## 3. AIV Ecology and Its Persistence and Infectivity under Various Environmental Conditions

To perform field monitoring of a virus circulating in a population most effectively, we need to possess reliable knowledge about the stability, transmission mechanisms, and infection potential of the viral particles in the environment. The influenza virion is a ribonucleoprotein particle covered by a lipid bilayer envelope. The fecal–oral route is the primary transmission route of avian influenza between birds [30,31]. The stability of viral particles in feces and freely within the environment (predominantly water) is a matter of highest importance in terms of virulence and in terms of field study, as well. The primary concern is the length of time the virus can persist and preserve its infectious capability under environmental conditions typical for biotopes where migratory fowl dwell and rest during travel. A classic 20th-century study on AIV in duck feces suggests that the viruses retain infectivity in fecal material for at least 30 days at 4 °C and for 7 days at 20 °C [32]. A field study in the northern wetlands of North America, namely Alaska, Minnesota, and Louisiana, combined with laboratory approaches estimated the virus’s viability in nature. AIV-containing paired cloacal and oropharyngeal swab samples from ducks were used to inoculate water samples, which were then stored in parallel in laboratory conditions and in the wetlands (the latter kept in perforated steel drums). The experiment lasted for more than 200 days and included the winter period. The authors revealed that virions may keep infectivity for more than seven months in surface waters obtained from Alaska and Minnesota field sites when kept under naturally occurring temperatures, although they showed a gradual decline in the number of samples with active virus detected over time. Physical conditions in the northern wetlands in which AIVs remained infectious included cold temperatures (approaching 0 °C) and near-neutral pH [33]. These results indicate that the aquatic environment, itself, is a prominent reservoir of AIV relevant for epizootiology. In terms of avian influenza field study, in the presence of sensitive methods such as SHERLOCK [34], the sampling of water from biotopes where migratory fowl dwell or rest could partially replace or supplement existing methods of sampling, such as pharyngeal and cloacal swabs and feces collection.

The interaction of waterborne AIV with invertebrate organisms that dwell in the biotope is an interesting area of research. Laboratory and field studies indicate that different mollusk and crustacean species are capable of affecting the viral titer in the water they inhabit. *Daphnia magna* water fleas have a tendency to accumulate AIV in their tissues, lowering the water titer, wherein the accumulated virions do not retain infectivity. Thus, the presence of water fleas in the biotope can be regarded as a factor reducing AIV dissemination [35,36]. Similar observations were made in freshwater snails *Physa* spp. [37], clams *Corbicula fluminea* [38], and mussels *Dreissena ploymorpha* [39]. In general, the mollusks reduce the amount of virus in the water by accumulating it within tissues, whereby the accumulated virus loses its infectivity. Similar results with virus accumulation in the tissues with concomitant depletion in water were shown for crayfish *Orconectes* sp. [40]. In contrast, freshwater shrimps *Atyopsis moluccensis* increase viral persistence in water [41]. In summary, most studied aquatic invertebrates will filter the virus out of the water, concentrating it in the tissue or otherwise disarming it. Therefore, such invertebrates may be regarded as indicator organisms for screening by means of RT-qPCR or in situ detection methods. Concurrently, their presence in the biotope acts as a factor reducing the virus distribution.

Another important characteristic of the aquatic environment is its pH. It is known from previous studies that AIV particles are stable and keep their infectious potential in the neutral or slightly alkaline pH range and infectivity decreases rapidly below pH 6.0 [42,43]. A recent comprehensive study recommends mixing fecal swabs exhibiting acidic pH with a neutral to light alkaline buffer medium of pH 7.0–7.5. Birds droppings from the *Anatidae*, *Laridae,* and *Phasianidae* families, which comprise most migratory waterfowl, show neutral to basic pH, so it is primarily birds from these families that act as natural reservoirs of AIV and are known to play a key role in AIV transmission. The neutral to basic pH of their feces could be responsible for the viability and efficient transmission of AIVs in species from these families [44].

Conversely, studies were designed to evaluate the persistence and infectivity of A/H5 HP AIV in slurry from various duck feces under neutral and alkaline pH conditions prepared by lime treatment. The viability and infectivity of the virus in slurry samples were estimated by viral replication in embryonated chicken eggs (see the section below). The virus persisted in slurry under neutral pH conditions from 2 to 7 weeks, depending on the strain, while the persistence of infectious A/H5 HP AIV in all slurry samples after lime treatment at pH 10 or pH 12 was under 1 week [45]. As such, it may be concluded that the optimal pH range for AIV persistence lies in the interval between 6 and 8, so the pH values of the storage media of AIV containing samples should be kept in this range. The primary conclusion of this overview of AIV persistence in the environment is that the virus is highly stable and retains infectivity for periods ranging from weeks to months. This knowledge can be used to improve the existing sampling and monitoring methodology and detect AIV directly in water by in situ methods followed by re-infection in test organisms.

## 4. Current Avian Influenza Virus Sampling, Analysis, and Quantification Methodology

The avian influenza virus is known to infect and proliferate primarily in the respiratory and digestive tracts [46]. As such, the typical ways to obtain samples of biological material containing the virus from wild migratory birds are (i) pharyngeal swabs, (ii) cloacal swabs taken from live or freshly killed birds, and (iii) collection of fresh bird feces and pellets. In addition, highly pathogenic AIV can also be detected in multiple organs in heavily diseased or deceased birds [47]. Fecal sampling allows the minimization of damage to the bird population and possesses by far a larger capacity than other methods, as fecal collection requires much less time investment and permissions than swabbing. Additionally, the collection of droppings is less cumbersome and does not require the permissions that are necessary for trapping or hunting birds [48]. Nevertheless, the probability of isolating a viable AIV sample from fecal biomaterial is, in general, lower than that from freshly taken tracheal and even cloacal swabs [47], so during the sampling of avian feces, special attention has to be paid to their quality in order to maximize the efficacy of live viral strain isolation. A study focusing on the dependence of AIV prevalence on environmental variables, such as bird quantity and species variety along with the temperature, rainfall, vegetation coverage, and water body size, has been performed in wetlands of Chile [49]. The results showed general prevalence of the virus in 4.28% of the droppings, with remarkable seasonal variations, peaking at 30% in summer months. Prevalence was positively associated with minimum temperature and negatively associated with water body size. Other studies in regions with similar, Mediterranean-like climates, such as Spain or Australia, have revealed close prevalence values ranging from 1.7 to 5.43% [50,51,52]. For regions with colder climates such as Alaska [53] or Mongolia [54], values of AIV prevalence in the fecal samples are comparable (1–7.3%).

The general approach to avian fecal sampling in such trials can be described as follows: The formula for counting required minimal sampling size is

$$n=\left(1-{\left(a\right)}^{\frac{1}{D}}\right)\left(N-\frac{D-1}{2}\right),$$

where *n* = required sampling size, *N* = population size, ∝ = 1 – confidence level, and *D* = estimated minimum number of positive samples [49].

In order to detect a prevalence of 1.5% [51] with 95% confidence, 178 samples need to be collected [49].

An intensive study in Alaska demonstrates an example where sampling was performed by both swabbing of hunter-harvested birds and feces collection, with additional classification of samples by host species, which is unusual for this type of study. The results allowed the researchers to distinguish between the impacts of different waterfowl species in AIV distribution and prevalence. It has been shown that even inside a given genus (Anas ducks) the prevalence may vary by orders of magnitude (from 25% to 2% or even 0%), thus detailing the picture of AIV epizootiology [53].

**Figure 1:**
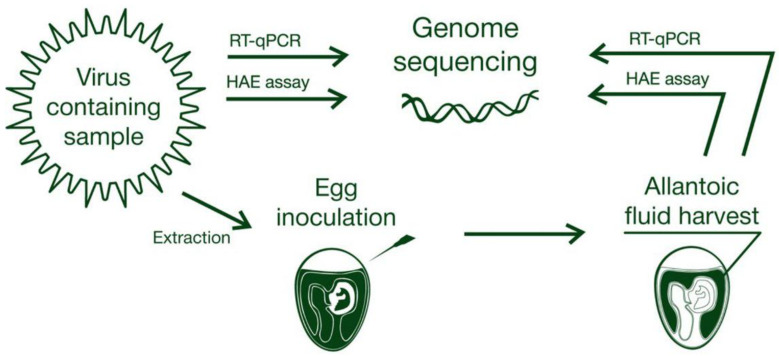
Standard protocol for AIV field sample analysis.

In general, there are two primary well-established methods to detect AIV presence in avian biomaterial, either feces or swabs, according to the OIE. The first is the inoculation of feces that were previously resuspended in a special antibiotic-containing medium, which is then centrifuged and filtered into fertilized chicken eggs. After subsequent incubation, allantoic fluids should be recovered and tested using a screening test, such as hemagglutination, or influenza A type-specific tests (agar gel immunodiffusion test, neuraminidase inhibition (NI) tests, and others). An alternative approach to the detection of AIV particles in the fluid is by use of molecular techniques to detect the presence of nucleic acid signatures specific to influenza A, such as real-time reverse transcription polymerase chain reaction (RT-PCR) testing [55], along with more specific assays developed for the simultaneous detection of different viral clades in field samples and in poultry [56,57]. It should be noted that, for large-scale screening experiments, the current strategy of viral reproduction in inoculated chicken embryos followed by a hemagglutination assay (Figure 1) is regarded as the primary method for viral detection, while RT-PCR and sequencing remain as means of final proof [54,58,59]. However, some studies use a reversed strategy, using RT-qPCR as primary screening method with subsequent egg inoculation of the samples testing positive (where a positive read means providing cycle threshold values ≤ 45) [53]. Currently, several established approaches to AIV diagnosis using RT-PCR and/or RT-qPCR coexist [57,58,59,60], which are adopted in most laboratories. The general strategy of these approaches is typically composed of initial generic detection of AIV in clinical or field specimens, primarily by targeting the most conservative matrix (M) gene segment, followed by specific RT-qPCR tests for different hemagglutinin subtype viruses. A comparative assay was performed, testing four frequently used laboratory assays and one commercially available kit for AIV detection, where both analytical sensitivity against eight different viral clusters and diagnostic performances against clinical specimens of known infectious status were evaluated [61]. The most frequently used target sequence for qPCR primers and probes is a 99 bp long region of the M gene (nucleotides 25-124). The study demonstrated that the best analytical and diagnostic performances were by recently developed protocols using degenerated primers [62,63], as opposed to older protocols with nondegenerate primers. This underlines the importance of monitoring AIV M gene segment evolution and the necessity of keeping primer and probe sequences up to date in relation to their binding regions.

A recent study described AIV sampling, detection, and isolation from bird feces in detail, drawing attention to the morphological, physiological, and biochemical characteristics of the samples in relation to their impact on the survival of AIV particles [44]. In the study, freshly gathered bird droppings of different taxa and in different physiological conditions were populated with AIV H9N2 at a 10^4^ 50% egg infectious dose (EID50) and incubated at room temperature (25 °C) for 24 h. After serial dilution, fertilized chicken eggs were inoculated. The primary conclusion of the paper was that AIV particles sustain relatively high viability and infectious potential at higher pH ranges (6–8), while more acidic pH inhibits the infective potential by an order of magnitude. Importantly, lowering the pH did not affect the efficacy of viral RNA detection by RT-PCR. Incubation of acidic feces in buffer with appropriate neutral to slightly alkaline pH (7.0–7.5) restored infectious virus titers (EID50) to higher values, so the authors recommend resuspending the biomaterial in an appropriate medium before proceeding with inoculation [44].

Occasionally, serum tests on AIV antibodies are used on blood samples from captured wild birds or domestic poultry. Serum samples are initially evaluated using a competitive enzyme-linked immunosorbent assay (c-ELISA) with the following hemagglutination inhibition (HI) test on samples positive for anti-AIV antibodies. Such testing allows for the distinction between viral serotypes. However, blood sampling of wild birds cannot be easily scaled up for screening, so this method is mostly applicable to studies conducted on poultry farms [64]. Meanwhile, more advanced strategies of AIV detection are coming into play. A colloidal gold immunochromatographic assay (GICA) was used for the rapid detection of influenza A (H7N9) and its efficacy has been compared to the traditional reverse transcription polymerase chain reaction (RT-PCR) and viral culture methods. Compared to RT-PCR, GICA demonstrated lower sensitivity (33.33%) but much higher specificity (97.56%). Compared to viral culture, GICA showed 91.67% sensitivity and 82.03% specificity. The GICA method showed the most detection efficiency in sputum specimens, as compared to samples from throat swabs and feces, with the sensitivity reaching up to 1 ng/mL for recombinant HA protein. GICA-based AIV testing is, in general, a reliable method for screening and diagnosis and can be used as an alternative to traditional methods, especially for preliminary diagnosis in early stages and resource-limited settings [65]. Another strategy for rapid detection of influenza in biomaterial samples is the use of fluorescent immunochromatography. An immunochromatographic strip test based on coumarin-derived dendrimers conjugated with latex beads has been developed. Its sensitivity allowed for the detection of 10 ng/μL of influenza A nucleoprotein antigen within 5 min, which corresponded to 2.5-fold higher sensitivity than that of the dot blot immunoassay. The assay is capable of recognizing at least four avian influenza A subtypes (H5N3, H7N1, H7N7, and H9N2) and of distinguishing them from other respiratory viral diseases. The test is also quantitative and uses an LED-based portable strip reader, which makes it applicable for field diagnostics [66]. A further improvement of this branch of methods involves the use of europium nanoparticles [67], artificially designed peptides specifically binding hemagglutinin [68], and improved fluorescent dyes [69]. Additionally, a smartphone-based immunofluorescent field diagnostic approach was described [70,71] and upgraded to a dual-fluorescent system [72] capable of specifically detecting the highly pathogenic H5 subtype. In this type of assay, a special portable fluorescent strip reader is required to develop the test strips, which are compatible with a smartphone camera. Although this type of test was optimized for clinical diagnosis in patients with H5 HPAI, it could also be applicable for field tests of avian biomaterial. Immunofluorescent methods continue to improve and to evolve towards nanoscale. A micellar nanoparticle-based immunoluminescent lateral flow assay has been recently developed, able to detect a panel of avian influenza viruses such as H3N2, H9N2, H1N1, and H5N9 with detection limits of 10^3.5^ EID50/mL, 10^2.5^ EID50/mL, and 10^4^ EID/mL, respectively [73].

It has to be mentioned that the novel generation of highly sensitive nucleic acid detection methods using CRISPR nucleases is gaining popularity for the supplementation or replacement of the previously mentioned traditional methods.

SHERLOCK [34] and its expansions and spin-offs, such as CARMEN-Cas13 [74], have already been tested and proven capable of detecting a multiplex array of viral pathogens simultaneously, and they are field-deployable [75]. This group of methods is based upon in situ isothermal amplification of nucleic acids such as recombinase polymerase amplification coupled with reverse transcription and T7 transcription to generate enough RNA substrate for the Cas13 nuclease, which is capable of recognizing specific sequences via complementary guide RNA. Following treatment of the amplified substrate by Cas13 results in its cleavage, along with collateral cleavage of a nonspecific RNA sensor in trans giving a fluorescent signal. Combining the programmable specificity of Cas13 with a reporter molecule that is activated upon target recognition, one can obtain a specific and sensitive indication of the presence or quantity of a given nucleic acid, such as the influenza virus RNA in the reviewed case [74,75]. As influenza virions possess long-term stability in water under natural conditions, considering that most avian feces are deposited in the water bodies and the main pathway of waterfowl infection is fecal–oral during feeding, we suggest a field-deployable technique based upon SHERLOCK and CARMEN methods. This would allow researchers to specifically detect various AIV strains in water samples in situ, as a replacement for the more tedious approaches of bird hunting and swabbing or feces collection and transport into the laboratory. Most obviously, the future of avian influenza monitoring in the coming decades will be tightly bound to the emerging technologies of this generation.

## 5. The Impact of Ecology and Migration Routes of Different Migratory Bird Taxa on the Dissemination of Different Avian Influenza Strains Worldwide

Waterfowl belonging to the *Anatidae* family (ducks, geese, and swans) are the primary reservoir of all 16 hemagglutinin and 9 neuraminidase subtypes of avian influenza viruses [30,76]. Migratory avian species inhabit a vast diversity of wetland and aquatic habitats, from rivers and lakes to the open ocean. Infected birds may indeed act as carriers of the virus, as ring recovery studies have shown migratory links between India, Europe, and Russia [77]. Data on the persistence of AIV in waterfowl populations have direct application in deciphering the epizootiology of these viruses in wild migratory fowl populations and, hence, in predicting and preventing disease outbreaks [78]. The role of *Anatidae* birds is confirmed by many studies worldwide. Most of the high-risk species in the list of birds potentially involved in the introduction of the HPAI H5N1 strain in Qinghai Lake—an important breeding and stopover site for waterfowl along the Central Asian Flyway—belonged to *Anatidae anseriformes*, accounting for over 39% of the total migration risk in spring and over 91% in autumn [79].

A series of comprehensive studies showed that, in the period between 2003 and 2012, regular transmission of the H5N1 HPAI virus occurred between migratory bird flyways, and avian migrations were correlated with the peaks of H5N1 HPAI epidemics. This suggests that the transmission of H5N1 HPAI is strongly associated with avian migration, which calls for increased collaboration between countries located along the same flyways [80]. However, a large-scale phylogeographic analysis reveals that AIV strain distribution and exchange within a given migratory pathway can be 4–13 times greater than the between-flyway exchange rate, thereby implying that migratory flyways play an important role in the structuring of AIV populations. This underlines the importance of the surveillance of rest site water reservoirs that intersect different flyways [81]. It is also important to note that there are studies stressing the role of domestic bird transportation and breeding as a dominating factor of avian flu distribution, as compared to the impact of migratory waterfowl [82]. Nevertheless, wild bird migration is a crucial facilitator of AIV dissemination across long distances, which allows for the introduction of new viral strains into areas with naive populations.

Detailed AIV monitoring studies, where avian biomaterial is collected in an area over the span of several years, are performed regularly worldwide and are necessary to reflect global influenza epizootiology. A recent five-year study in Alaska was focused on certain species causing a major impact on AIV distribution in the region, namely dabbling ducks (*Anas* spp.), emperor geese (*Chen canagica*), and glaucous-winged gulls (*Larus glaucescens*). The study revealed little evidence for differences in viral subtypes and diversity between different waterfowl hosts; however, subtypes and viral diversity varied between waterfowl host groups and glaucous-winged gulls. Interestingly, sequencing and bioinformatic analysis revealed that a higher percentage of viral sequences from northern pintails (*Anas acuta*), emperor geese, and glaucous-winged gulls were grouped in phylogenetic clades that included AIV sequences originating from wild avian species sampled in Asia, as compared to non-pintail dabbling ducks. This difference most likely emerged due to intercontinental migratory tendencies of the host species, indicating ducks, geese, and glaucous-winged gulls are more significant AIV shuttles between continents than non-pintail ducks [53].

Similar long-term studies have been performed on the Asian continent by the same research groups in Japan (2000–2007 and 2010–2014) and Mongolia (2010–2014 and 2017–2019), where sampling was done by fecal collection [54,83,84]. No differential analysis between host species was performed, but a detailed viral sequence analysis was done, revealing in total, 15 H5 viruses, including 2 HPAIVs, and 25 H7 influenza A viruses were identified and characterized during these two studies.

The antigenic analysis of the isolated viruses showed the high stability of the antigenicity of viruses in wild aquatic birds, which was dependent on their nucleotide sequence diversity and distinct from that of HPAIVs isolated in Asia in the same time period [54,84]. The authors concluded that the HPAIVs and Chinese H7N9 viruses responsible for avian influenza outbreaks in Asia during the period of study were not predominantly persisting in migratory waterfowl in Mongolia and Northern Japan, aside from sporadic introductions. However, they recommend continued monitoring of H5 and H7 influenza viruses, both in domestic and wild birds, for the control of the disease.

Another multidecade study was performed in North America between 1976 and 2015. The samples were sourced from migratory waterfowl throughout the Central and Mississippi Migratory Flyways in the United States and Canada. As the sampling was exclusively done by swabbing of live-caught or hunter-harvested birds, it was possible to correlate the species and the isolated viral strain. AIVs were detected in 8427 (0.8%) of 77,969 samples. A total of 96 hemagglutinin (HA)/neuraminidase (NA) subtype combinations were isolated, which included most HA (H1 to H14) and all 9 NA subtypes. Notably, dabbling ducks accounted for 91% of all samples, confirming the postulate of *Anatidae* birds being the main reservoir and transmitter of the disease. Annual trends of influenza prevalence and diversification have been observed. A few dominant subtypes were detected on northern breeding grounds during the summer at high prevalence. The prevalence of these strains lowered progressively as the waterfowl migrated toward southern wintering grounds, along with an increase in subtype diversity. This finding may suggest that increased diversification of strains is causing a decrease in the prevalence of dominant strains—a dilution-like effect. The authors also note that the driving force of this phenomenon may be the maturation of immune systems of juvenile waterfowl through interactions with new populations. Notably, isolates obtained during winter had the highest percentage of mixed and rare HA/NA combinations, providing an increased opportunity for AIV reassortment. In addition, 70% of H5 and 49% of H7 AIV isolates (which represent the main danger for human transmission) were obtained from samples collected during fall and spring, respectively; these are subtypes that can have significant implications for public health and agriculture sectors [85].

It was found that wild waterfowl migrating along coastal areas of the East Asian–Australasian Flyway demonstrated a high degree of variability in both resting locations and distribution of breeding areas. Several Eurasian wigeons and northern pintails settled in areas up to thousands of kilometers south of their central reported breeding area in Siberia. The use of breeding grounds in lower latitudes, where avian influenza is more common [86], could have significant implications on the diversity of viral strains transmitted on wintering grounds [43,87], as well as the general outbreak potential. The two diverse migratory strategies by long-distance and intermediate-distance migrants from the same wintering area were consistent with the findings [87] reported for mallards in California. Observations indicate that species-level differences in migration timing and behavior for common and widespread waterfowl species need to be considered, along with their unique temporal and spatial migration strategy, when incorporating wild birds into AIV risk modeling, prediction, and management [88]. Another analysis, focusing on large-scale geographical spread of the H5N8 HPAI virus and based upon four different sources of data, indicated that the main routes of viral dissemination were bound to long-distance flights of infected migratory wild birds, first in the spring of 2014 from South Korea, or other unsampled locations in the region, to northern breeding grounds and, in the autumn of 2014, further on to wintering sites in North America and Europe [89].

The role of wild birds in the spread of H5N1 HPAI has previously been investigated by comparing the patterns of disease spread to bird migration routes. However, until recently, the differing influence the southward autumn and northward spring migration may play in virus dissemination had yet to be assessed. H5N1 HPAI transmission was studied by analysis of direction and angular concentration of currently circulating viral clades, and compared to waterfowl seasonal migration paths along main waterfowl flyways. Four northward transmission directions were found along Asian flyways, where the initial epicenter of the virus was located in Southern China, where the H5N1 HPAI virus was first discovered in poultry [90]. The authors suggested that waterfowl were first infected by the virus in East Asia and then carried it north during spring migration, later spreading it to other parts of the world, primarily during autumn migration. Overall, the authors claim that the autumn migration of waterfowl plays a more significant role in the transmission of HPAI H5N1 compared to spring migration [91]. This is logical, as birds migrating in autumn include the novel summer-born generation, which is immunologically naive and thus especially susceptible to AIV infection.

The search for novel reassortant AIV strains is an ongoing process; as such, two H3N6 viruses were isolated from swan geese in Poyang Lake, Jiangxi Province, China, in 2014. Phylogenetic analyses indicated that these strains were recombinants of Eurasian-originated H3Ny (N3, N6, N8) and H5N6 viruses circulating among both wildfowl and poultry. Notably, H9N2 viruses have contributed their PB1 gene to these novel H3N6 viruses, illustrating the constant viral reassortment in the wild bird population worldwide [92]. An H2N9 LPAI strain was recently discovered in Korea, being the first reported in this country [93]. Its phylogenetic analysis revealed a complex origin from both Eurasian and East Asian viruses.

Satellite-based tracking of migratory waterfowl is an important, if costly, tool for estimating the role of wild birds in the long-distance transfer of avian influenza. An alternative indirect tracking method that utilizes stable isotope ratios in the feathers of migrating birds has shown potential in a study on migratory ducks in Asia. The authors recommend its use in combination with satellite tracking for achieving optimal efficiency at lower costs [94].

Avian influenza analysis can be assisted by modern mapping tools that can overlay various risk factors onto a map, including strain occurrence. There are various mapping tools with this capability, such as geographic information systems (GIS), the Influenza Research Database (IRD) [95], OpenFlu [96], and EMPRES-i [97]. A combination of phylogenetic analysis and mapping of biomaterial sampling has revealed the links between the evolutionary and geographical patterns of influenza A (H7N9) infections, where cases in the same or nearby municipality/provinces were grouped together with minor evolutionary differences. Moreover, three epizootic waves in domestic chicken populations along the East Asian–Australasian flyway in China could be distinguished from the obtained phylogenetic tree. GIS made it possible to identify potential migratory bird species as candidates responsible for the H7N9 infections in different Chinese provinces, such as Shanghai, Jiangsu, Zhejiang, Fujian, Jiangxi, and Guangdong [98] which account for 91.2% of the total number of isolated H7N9 cases in chickens in the global influenza database (GISAID). Monitoring the H7N9 strain is particularly important, as it is responsible for multiple waves of human infection in China in the past decade [99]. Based on the spatial distribution of the identified bird species, geographical AIV hotspots are further positioned and charted within typical municipalities/provinces [98].

A detailed study focusing on wild ducks and gulls as the major reservoirs for avian influenza A viruses was conducted in the Republic of Georgia, which is located at the intersection of three migratory flyways: the Central Asian flyway, the East Africa/West Asia flyway, and the Black Sea/Mediterranean flyway. Such regions serve as hotspots for emerging novel AIV strains through complex evolutionary mechanisms at sites where various duck and gull species from multiple flyways breed, winter, or stage. For six years (2010 to 2016), AIV samples from various duck and gull species that breed, migrate, and overwinter in Georgia were collected. The sampling was primarily performed by catching live birds and taking paired oropharyngeal and cloacal swab specimens, serum specimens, and, in some cases, feather samples. Twenty-four HA/NA subtypes of influenza A virus, including 12 different HA subtypes (H1–H7, H9, H10, H11, H13, and H16), were isolated. The value of this study is in the precise identification of a bird host species for each sample taken and in the confirmation of the fact that ducks and gulls are, indeed, the primary reservoirs and transmitters of the virus in the region. The study demonstrated a substantial subtype diversity of viruses (primarily LPAI) that varied in prevalence from year to year. Two HPAI strains belonging to clade 2.3.4.4, H5N5 and H5N8, were also identified. The authors concluded that the diffusion of avian influenza viruses within a multihost ecosystem is heterogeneous and, within a single host group (i.e., duck or gull species), cannot be used as a substitute for others. The evolution of the influenza viruses in their natural biotopes is a complex mix of host–pathogen interactions and ecological factors. Understanding the driving forces of viral evolution is key to investigating the emerging pathogens, interpreting the data from different sites around the world, and, ultimately, understanding the risk of incursion of emerging variants from one geographic region to another [100].

The claim that wild migratory birds are able to transmit the HPAI viruses over long distances unhampered and remaining asymptomatic was critically assessed by several studies, demonstrating that long-distance migration leads to immunosuppression and that migratory performance is negatively affected by infections. These findings place the idea that wildfowl can spread the virus along established long-distance migration routes into question [101].

Nevertheless, infected, symptomatic wild birds may act as transmitters over shorter distances, as appears to have occurred in Europe in early 2006 [101]. When wild birds migrate, they pass the virus along the flyway between individuals, so that it may travel long distances, so it would not be singular individuals transporting the virus for long-distance flights.

While most studies have focused on aquatic wild migratory birds, as they serve as both reservoirs and dissemination agents of avian influenza, some surveys have revealed an important prevalence of AIV among land birds as well. Recent comparison studies of the prevalence of influenza A viruses in different groups of wild birds (waterbirds and land birds; resident and migratory) in eastern Mexico, a place of convergence of the three main North American migration flyways, reveal an overall high prevalence of 39%. Surprisingly, prevalence was particularly high in land birds (49%) as compared to aquatic birds (26%), and there was no difference in overall prevalence between resident (39%) and migratory birds (39%) [102]. According to this study, the reservoir potential of land birds can be even higher than that of the waterfowl, and this has to be taken into account in monitoring studies. However, it has to be considered that the authors used an unusual method of capturing live animals and collecting their feces while holding them captive in the case of land birds, while data on waterfowl were obtained by hunter-harvesting, which could lead to a certain bias in numbers when compared to standard methods of swabbing and/or stool collection. Despite this, in places where the role of migratory waterfowl as long-distance transmitters of the virus is doubted, one could arrive at a model where migratory birds spread the virus through shorter distances, while land birds serve as AIV reservoir throughout the entire migration route, allowing multiple infection–transmission acts during the migration process.

To summarize, it can be claimed that there are certain taxa of migratory birds, predominantly ducks, followed by geese and gulls, that are primarily responsible for annual AIV dissemination and for the occurrence of novel strains through reassortment. The primary sources of knowledge about the emergence and distribution of AIV in time and space are long-term field studies (from several years to decades), where the virus-containing biomaterial is collected and analyzed, providing data for GISAID, the world influenza database, which later provides the ability to track the history of any known AIV strain [103]. Technical methods, such as geoinformatic systems, satellite tracking of birds, or analysis of feather isotopes, add an additional dimension to global avian influenza monitoring. Surmising from GISAID and long-term field study data in migratory birds, we are able to come to the conclusion that some regions have been and continue to be monitored quite regularly and precisely, among them North America, Europe, and Southeastern Asia. Conversely, there is a lack of data on Russia and, particularly, Siberia, which can be explained by low population density, harsh climatic conditions, and the depressed economic situation in this region. Nevertheless, the role of Siberia for avian influenza monitoring is hard to underestimate, as it is the region where migratory waterfowl and other birds from Southeastern Asia and Africa reside in summer, breeding and cross-contaminating each other with different AIV strains, and where multiple migration flyways cross, leading to intercontinental dissemination of the disease. This can be affirmed by the fact that an H5N8 HPAI strain was detected in Tyva, located in southern Siberia along the Central Asian Flyway, in 2016. The virus was detected in several species of waterbird: six black-headed gulls (*Larus ridibundus*), four grey herons (*Ardea cinerea*), four great cormorants (*Phalacrocorax carbo*), one great-crested grebe (*Podiceps cristatus*), one common tern (*Sterna hirundo*), and one duck (family *Anatidae*; species unidentified) [104]; the presence of an HPAI strain in the area demonstrates that Siberia is an important place to monitor. Long-term field studies of AIV in migrating birds in regions that are scarcely monitored should be encouraged so that awareness of the global situation may improve.

## 6. Interaction of Wild Migratory Fowl with Other Influenza Reservoirs such as Livestock, Food Markets, and Poultry Farms

AIV dissemination and evolution monitoring in migratory wildfowl would provide incomplete results if the other vast and important AIV reservoir is ignored, namely poultry farms and markets. Poultry markets, especially in Asian countries, are a form of “bioreactor”, where dozens of alive and freshly slain animals are brought together, providing an excellent platform for the viral strains infecting these organisms to establish contact and recombine. Consequently, regular AIV monitoring in domestic poultry is essential to provide the necessary context to the wildlife studies and give a complete picture of the zoonosis.

Hence, there is a certain need for new approaches to improve the quantification of the dynamic interaction and exchange of viral strains between wild fowl hosts and domestic poultry.

An outbreak of HPAI virus of the H5 A/goose/Guangdong/1/96 lineage mentioned in the second section of this manuscript has resulted in spillover from Asian domestic poultry into wild birds and spread, via waterfowl migration, to countries in Europe, Africa, and North America, making the outbreak global. In 2016/2017, this spillover resulted in the greatest HPAI epidemic recorded in Europe. A peculiarity of the outbreak is that it was associated with an unusually frequent reassortment rate between H5 HPAI viruses and cocirculating low pathogenic avian influenza viruses, leading to recombinations of viral gene segments typical for viruses persisting in domestic and wild anseriform bird populations. In addition to domestic Anseriformes in Asia, both migratory wild birds and domestic Anseriformes in Europe are relevant sources of gene segments for the ongoing reassortment of H5 HPAI viruses. The simplicity of the H5 HPAI virus reassortment, in combination with iterative spillovers of H5 HPAI viruses into wild fowl, heightens the risk of emergence of a recombinant virus that persists in wild bird populations yet remains highly pathogenic for poultry [105]. Research has determined that the outbreak was coupled with a change in the tissue tropism of the virus. It was found that 2016 H5N8 had acquired enterotropism, similar to LPAI viruses, without losing the respirotropism of older HPAI viruses in the Goose/Guangdong lineage. This permitted the virus to retain high pathogenicity combined with efficient infectivity through the fecal–oral route and an increased chance to persist long term in the wild waterbird reservoir [106].

Another spatiotemporal analysis of virus diffusion patterns during three H5Nx HPAI intercontinental epizootic waves was performed, which demonstrated that Africa mainly acted as an ecological sink for the viral pool of these strains. A combined analysis of host dynamics and continuous spatial diffusion indicates that both poultry trade and wild bird migrations have contributed to the virus spreading into Africa, with West Africa acting as a critical hotspot for virus introduction and dissemination into the continent [107].

Studies on satellite-marked waterfowl, namely blue-winged teal (*Anas discors*), were performed, with Bayesian joint hierarchical modeling being applied to emulate species distributions, residency times, migration periods, and disease outbreak probability under an integrated bird migration and disease distribution modeling framework. The results demonstrated that migratory waterfowl are correlated with AI occurrence within North America, showing a positive correlation between waterfowl residence time at a given location and the probability of a commercial poultry influenza outbreak in the location. Remarkably, AIV occurrence probability is higher during observed waterfowl northward migration and lower during the southward migration. The authors claim that this can be specific to the chosen model duck species and that, for other types of waterfowl, a reverse regularity would be more prominent due to increased hatchling abundances and bird aggregation at premigration staging areas through the late-breeding and fall seasons [108].

Another study linking the HPAI distribution in poultry and among wild migratory birds was performed in the Netherlands in 2016–2017. The densities of the selected HPAI high-risk wild bird species, such as Eurasian wigeons, tufted ducks, *Anatidae* (ducks, geese, and swans), and *Laridae* (gulls), around H5N8 HPAIV-infected farms were compared to the densities around noninfected reference farms, also taking the altitude and amount of aquatic bodies into account. A positive correlation between HPAI-infected farms and migratory waterfowl density was observed, confirming the hypothesis of instant pathogen exchange between these two types of AIV reservoirs [109].

Between 2008 and 2010, H5N1 was brought into West Bengal at least three times, as shown by cluster analysis. Poultry farms in areas that intersected habitat and resting sites of wild birds had a much higher chance of suffering outbreaks. A stochastic mathematical model was used to simulate the introduction of H5N1 to local poultry through contact with wild migratory fowl, which demonstrated that reduced contact between wild birds and domestic poultry and increased culling rate of domestic birds that are infected would reduce the likelihood of outbreaks [110].

Research on AIV diversity in live poultry markets (LPMs) was performed between 2015 and 2016 in 10 LPMs in the Hubei, Zhejiang, and Jiangxi provinces, China. Strikingly, the researchers observed not only high diversity and prevalence of AIVs (totaling 12 subtypes) but also a drastic difference in the detected strains as compared to those reported by the same team in the same locations one year previously, suggesting a dynamic shift in genetic diversity of the virus over the course of a single year. Phylogenetic analyses revealed frequent reassortment, including reassortment between LPAIV and HPAIV subtypes and between strains circulating in domestic and wild fowl. Importantly, the novel H5N6 reassortant strain, which contained a set of H9N2-like internal genes, was prevalent in all three regions inspected. Overall, these data highlight the extensive changes in genetic diversity and in recombination patterns in the AIVs that circulate in LPMs [80].

Studies on Mongolian wild bird AIVs suggest that strains isolated by the researchers have evolved with multiple genotypes and are closely related to poultry AIVs, as well those in wild Korean birds, thus confirming the hypothesis of intense gene exchange between strains in neighboring areas and between poultry and wild fowl [111].

Another large-scale study focused on the role of the live poultry trade in the spread and maintenance of HPAIV in Asia. The authors combined virus genomes, reconstructed poultry transportation data, and compared the spatial spread of three key subtypes of AIV (H5N1, H7N9, and H5N6) throughout China to estimate the impact of the poultry trade in disseminating AIV over large geographic areas in developing countries with complex poultry production systems. They managed to separate the role of poultry trade from other disseminating factors, such as bird migration, and determined that the dissemination of these subtypes among domestic poultry is geographically continuous and likely associated with the intensity of the live poultry trade in China. Using two independent data sources and network analysis methods, the authors reported a regional-scale community structure in China that could be useful for future monitoring of the spread of AIV subtypes in the country. The identification of this structure, supplemented with data on viral spread through wild bird migration, has the potential to develop more targeted strategies for the prevention and control of AIV in China [112].

Additionally, it is important to consider a North American study that found virus exchange between domestic poultry and wild migratory fowl populations could occur through airborne matter. The unprecedented H5N2 HPAI outbreaks in the United States in 2015 devastated poultry production and resulted in over USD 3 billion of economic losses. It was reported that, in some cases, the AI virus was aerially delivered into poultry houses, as abnormal bird mortality was recorded near air inlets of infected houses. This study modeled air movement trajectories and virus concentrations that were used to assess the probability or risk of airborne transmission for the 77 HPAI cases in Iowa. The probability of airborne HPAI infection was found to be affected by farm type, flock size, and distance to previously infected farms. More significantly, it was markedly reduced by swift depopulation and inlet air filtration. These results provide insight into the risk of airborne transmission of HPAI virus via fine dust particles and the importance of preventative and containment strategies, such as air filtration and quick depopulation of infected flocks [113].

Transmission of the virus to domestic poultry has a huge impact on both commerce and human safety, as has been discussed throughout this paper. However, the risk of the interspecies infection of nonhuman mammals also needs to be underlined, as AIV has been known to affect farmed swine and, through them, humans. A study on LPAI strains of the H7 subtype, which are infrequently, but persistently, associated with outbreaks in poultry in North America, revealed that the given strains have developed enhanced virulence and transmissibility towards mammals. H7 AIVs have resulted in both occasional acquisition of an HPAI phenotype in birds and sporadic cases of human infection. Laboratory studies have shown the ability of H7 LPAI strains to efficiently infect and propagate in mouse and ferret models, confirming the overall constant risk of AIV transmission to mammals and the need for constant monitoring of the virus in nature [114]. To summarize, contact between poultry farms and migrating waterfowl is a pronounced risk factor for AIV dissemination and reassortment. As this virus has been known to infect nonhuman mammals, such as swine, precaution to prevent AIV may be beneficial in nonfowl farmed animals. Extra attention has to be paid to prevent contamination of farms lying on the migration flyways of wild birds, as such contamination can occur even in airborne matter. More intense poultry vaccination in such hotspots could be a major safety benefit.

## 7. Monitoring and Bioinformatic Prediction of AIV Strains with Zoonotic Potential

Despite the plethora of strains circulating in the wild avian population and poultry, only a handful give rise to zoonotic influenza outbreaks that show the potential to infect humans. There are natural obstacles between species which prevent influenza strains from jumping to another host species easily. For example, host proteins of the ANP32 family are essential for influenza polymerase complex activity [115]. However, in different species, different members of the family are utilized by the virus. Avian influenza strains are mostly dependent on ANP32A, while mammalian strains tend to use ANP32B, making these proteins an interspecies barrier [116,117]. On occasion, however, complex influenza reassortants arise, combining avian, swine, and human features, gaining the ability to infect multiple classes of animal and being extremely dangerous as potential zoonotic pathogens because of it [118]. Long-term AIV field studies give input to detailed information on the global situation, where all possible strains detected are mapped by location and timescale. Such data are available on GISAID—a global scientific initiative and primary source that provides open access to genomic data of influenza viruses [103]. This brings up the question of whether it is possible to predict a newly identified strain as zoonotic based upon its genomic features and on the pool of previously known information. Different viral strains possess diversely expressed tropisms to different species. Several bioinformatic approaches were applied to predict the tropism of an influenza virus strain, relying on its genomic sequence or proteomic composition. This was used to make a conclusion on whether the given strain was prone to infecting humans and being zoonotic. Zoonotic strains typically arise from mutations or reassortment, granting them the capability to avoid the host species barrier and successfully infect a new host. Phylogenetic analyses and genetic markers are useful in tracing the origins of zoonotic infections, but there are still no effective tools to identify high-risk strains in advance, prior to the outbreak occurring. One of the modern methods is the identification of host tropism protein patterns or signatures that are highly likely to emerge in zoonotic strains. It exploits the high-accuracy classification of influenza A tropism based on input features that characterize amino acid frequency and composition, as well as the biochemical properties of amino acids, such as transition and distribution features for the hydrophobicity, normalized van der Waals volume, polarity, polarizability, charge, and solvent accessibility. A machine learning-based bioinformatic analysis of previously known human, avian, and zoonotic influenza viruses has helped to reveal certain mixed patterns of proteins, partially typical for human and avian hosts. Briefly, an avian strain carrying a certain percentage and/or pattern of human-strain-related proteins is considered to be potentially zoonotic. The authors describe their approach as a tool for the evaluation of the zoonotic potential of future AIV strains [119,120].

Convolutional neural networks gave rise to an extension of this approach, another model for classifying a given influenza strain or protein as human, avian, or zoonotic and highlighting crucial tropism-relevant amino acids on 3D protein models [121].

Another study addressed the same problem, defining AIV human adaptation as the capability to infect humans easily, to transmit among populations efficiently, and to be virulent to some degree in humans. The authors used machine learning-based bioinformatic analysis to reveal specific dinucleotide compositions usable as fingerprints for the human adaptation of avian viruses. They succeeded in predicting the human adaptation of the swine/avian AIVs before and after the 2009 H1N1 pandemic and in the identification of the human adaptation-associated genomic composition of AIV segments. Machine learning models using large AIV genomic data sets pave the way for the identification of key viral factors that affect virus transmission/pathogenicity and for the prediction of pandemic influenza [122].

A bioinformatic approach named host-prediction-based probability estimation of reassortment (HopPER) estimates the reassortment probabilities of influenza viruses through host tropism prediction using 147 new features generated from seven physicochemical properties of amino acids [123].

This group of methods allows users to apply neural networks to calculate the most relevant amino acids or nucleotide distribution patterns for tropism. While influenza is known for its broad host tropism, insufficient data exist on the species tropism of certain viral strains. Currently, the virulence of new virus strains is unknown. Mining the databases of past circulating strains, machine learning methods may be the key to estimating the virulence of newly discovered ones.

## 8. Conclusions

Avian influenza remains one of the major sources of epidemics and epizootic outbreaks worldwide, causing annual financial losses of billions of dollars due to domestic poultry culls and being responsible for human mortality. The high variability of the virus makes it impossible to create a vaccine with a long-lasting effect. The source of the high variability is the vast reservoir represented by the wild bird population, where migratory waterfowl, primarily representatives of the *Anatidae* family, play the role of major carriers of novel, potentially highly pathogenic, virus strains throughout various habitats via the avian migratory pathways. The emergence and evolution of new HPAI strains are more prevalent within certain avian migration pathways. Therefore, regions where several migration pathways intersect are highly significant when it comes to viral evolution by means of the genetic shuffling of several strains infecting the same host. Annual monitoring for novel avian influenza strains and the prediction of potential HPAI outbreaks is particularly crucial in these areas. Among them, Russia, especially Siberia, is understudied. A recent outbreak of the H5N8 strain of influenza on a poultry farm in the Astrakhan region of Russia resulted in the first human infection with the strain ever reported [29]. The African continent is similarly understudied, with one of the most recent large-scale studies of the virus conducted in 2017, with only 23 of 54 countries participating. In the 43 weeks of the study, 29,500 specimens were collected, and, of them, 28,441 were tested; 4300 samples tested positive—a prevalence of 15% [124]. Continued monitoring on the continent is highly relevant; a poultry farm that had been affected by the H5N8 HPAI outbreak in Mpumalanga Province, South Africa, in 2017, has once again been affected by this strain. This was discovered on 13 April 2021, after 300 birds died and tested positive for the strain [27,125]. These events underline the imminent threat of avian influenza zoonoses and the importance of monitoring the spread of the virus, particularly in countries that are underrepresented in studies and lie along the migration flyways of wild birds, as many parts of America, Asia, and Europe are extremely well studied.

Given the overview of existing and developing methodologies, we can propose further development of strategies for performing avian influenza and HPAI zoonosis outbreak monitoring and prevention.

**Figure 2:**
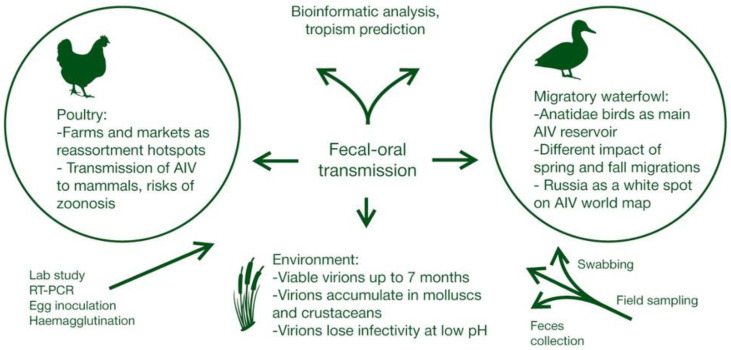
Avian influenza virus ecology and research.

First of all, regular expeditions into regions important from the point of view of waterfowl migration (such as large bodies of water located along major flyways, where the birds take their rest) have to be performed. We already mentioned regions that are understudied from this point of view, but sampling in well-studied regions such as Southeastern Asia has to be continued as well. Field expeditions should not limit their sampling options to traditional approaches, which are often tedious or harmful for the environment. Soon they will be equipped with up-to-date field-deployable systems of novel viral strain detection, such as the extremely sensitive methods using CRISPR nucleases or immunofluorescent chromatographic strips. Given the month-long stability of infectious AIV particles in water, such novel highly sensitive field screening methods should soon come into play, whereby the virus is detected directly through water probes, avoiding the procedures of hunting, swabbing, or feces collection. Novel field-deployable screening methods will be efficiently combined with the fast-developing GIS technologies to enable the real-time tracking of emerging AIV strains. Such 21st-century approaches will allow us to keep potential outbreaks of avian influenza in check and to increase the knowledge about its spread, evolution, and pathogenesis (Figure 2). Data on novel viral strains will be used for the development of novel vaccines using the reverse genetic systems. Moreover, collections of biomaterial obtained in field expeditions can be analyzed on multiplex platforms such as CARMEN-Cas13 [74] for the characterization of not merely influenza strains, but entire pathogenic viromes, thus making the global monitoring multidimensional.

## Data Availability

Not Applicable.
